# Detecting localised prostate cancer using radiomic features in PSMA PET and multiparametric MRI for biologically targeted radiation therapy

**DOI:** 10.1186/s13550-023-00984-5

**Published:** 2023-04-26

**Authors:** Tsz Him Chan, Annette Haworth, Alan Wang, Mahyar Osanlouy, Scott Williams, Catherine Mitchell, Michael S. Hofman, Rodney J. Hicks, Declan G. Murphy, Hayley M. Reynolds

**Affiliations:** 1 Auckland Bioengineering Institute, The University of Auckland, Auckland, New Zealand; 2 Institute of Medical Physics, School of Physics, The University of Sydney, Sydney, NSW Australia; 3 Centre for Medical Imaging, Faculty of Medical and Health Sciences, University of Auckland, Auckland, New Zealand; 4 Centre for Brain Research, The University of Auckland, Auckland, New Zealand; 5 Sir Peter MacCallum Department of Oncology, The University of Melbourne, Melbourne, VIC Australia; 6 Division of Radiation Oncology, Peter MacCallum Cancer Centre, Melbourne, VIC Australia; 7 Department of Pathology, Peter MacCallum Cancer Centre, Melbourne, VIC Australia; 8 Cancer Imaging, Peter MacCallum Cancer Centre, Melbourne, VIC Australia; 9 Department of Medicine, St Vincent’s Hospital Medical School, The University of Melbourne, Melbourne, VIC Australia; 10 Division of Cancer Surgery, Peter MacCallum Cancer Centre, Melbourne, VIC Australia

**Keywords:** Multiparametric MRI, Prostate cancer, PSMA PET/CT, Radiomics, Radiation therapy

## Abstract

**Background:**

Prostate-Specific Membrane Antigen (PSMA) PET/CT and multiparametric MRI (mpMRI) are well-established modalities for identifying intra-prostatic lesions (IPLs) in localised prostate cancer. This study aimed to investigate the use of PSMA PET/CT and mpMRI for biologically targeted radiation therapy treatment planning by: (1) analysing the relationship between imaging parameters at a voxel-wise level and (2) assessing the performance of radiomic-based machine learning models to predict tumour location and grade.

**Methods:**

PSMA PET/CT and mpMRI data from 19 prostate cancer patients were co-registered with whole-mount histopathology using an established registration framework. Apparent Diffusion Coefficient (ADC) maps were computed from DWI and semi-quantitative and quantitative parameters from DCE MRI. Voxel-wise correlation analysis was conducted between mpMRI parameters and PET Standardised Uptake Value (SUV) for all tumour voxels. Classification models were built using radiomic and clinical features to predict IPLs at a voxel level and then classified further into high-grade or low-grade voxels.

**Results:**

Perfusion parameters from DCE MRI were more highly correlated with PET SUV than ADC or T2w. IPLs were best detected with a Random Forest Classifier using radiomic features from PET and mpMRI rather than either modality alone (sensitivity, specificity and area under the curve of 0.842, 0.804 and 0.890, respectively). The tumour grading model had an overall accuracy ranging from 0.671 to 0.992.

**Conclusions:**

Machine learning classifiers using radiomic features from PSMA PET and mpMRI show promise for predicting IPLs and differentiating between high-grade and low-grade disease, which could be used to inform biologically targeted radiation therapy planning.

**Supplementary Information:**

The online version contains supplementary material available at 10.1186/s13550-023-00984-5.

## Background

Prostate cancer is the second most common cancer in men worldwide after lung cancer and the fourth most common cause of cancer death [1]. Traditionally, patients with localised prostate cancer who have radiation therapy receive a uniform dose to the entire gland, which is planned and delivered using CT imaging. Prostate cancer is known to be a multi-focal disease, however, and intra-prostatic lesions (IPLs) are not typically visible on CT. To address this, multiparametric MRI (mpMRI) and/or Prostate-Specific Membrane Antigen (PSMA) PET/CT can be used to assist in defining IPLs and have been used to inform targeted boost-focal radiation therapy planning. Such boost-focal strategies have been shown to improve biochemical control rates compared to traditional uniform dose prescriptions [2].

An alternative focal dose boosting approach is Biologically targeted Radiation Therapy (BiRT) which aims to deliver a non-uniform radiation dose distribution to the tumour according to its spatially defined biological characteristics [3]. The BiRT approach aims to eradicate the tumour while minimising radiation exposure to normal tissue, which requires an accurate delineation of the tumour at a voxel-wise level and an estimation of the tumour characteristics. This information can be derived from imaging to devise an optimised, tailored dose distribution [3].

Combining mpMRI and PSMA PET/CT to implement BiRT offers advantages to using either modality alone, as they offer different but complementary information. Multiparametric MRI provides anatomical information from T2-weighted imaging and functional information from diffusion-weighted imaging (DWI) and dynamic contrast enhanced (DCE) MRI which characterise tumour diffusion and perfusion, respectively [4]. PSMA PET using the Ga68-PSMA-11 tracer offers molecular information by indicating its level of PSMA type II transmembrane protein expression [5]. PSMA PET has shown high detection sensitivity especially for high-risk disease [6, 7]. Using PSMA PET and mpMRI together reduces the drawbacks of each, with mpMRI at times missing small or low-grade tumours [8], whereas PSMA PET suffers from low spatial resolution, can struggle to visualise tumours which are close to the bladder base due to urinary activity, and a small proportion of tumours do not express PSMA. The benefits of using mpMRI and PSMA PET together were analysed in the recent multicentre PRIMARY clinical trial, showing that it reduced false positives when detecting clinically significant disease compared with mpMRI alone [9].

Despite these benefits, there are still uncertainties regarding how signals in PSMA PET and mpMRI compare with each other, owing to the limited number of studies and lack of ground truth histology data to quantify their relationships. Furthermore, to facilitate non-uniform IPL dose-distribution approaches such as BiRT, a voxel-wise approach must be taken. Hence, this study aimed to assess the relative value of mpMRI and PSMA PET at a voxel level, by building upon our earlier proof-of-concept study [10], and by investigating machine learning models incorporating radiomic features to detect IPLs and predict grade, which could be used to inform BiRT treatment planning.

## Methods

### Patient data

Data from 19 patients diagnosed with localised prostate cancer and recruited as part of a Human Research Ethics Committee (HREC)-approved study (HREC/15/PMCC125) were included. Patients were scheduled for radical prostatectomy at the Peter MacCallum Cancer Center in Melbourne, Australia, and had mpMRI and PET/CT imaging prior to surgery. Patient clinical, imaging, and pathological details are given in Table 1. The Prostate Imaging-Reporting and Data System (PI-RADS) for the index lesion on mpMRI ranged from 2 to 5, with patient 59 being indeterminate due to imaging artefacts. SUVmax on PSMA PET ranged from 2.43 to 59.40. The Grade Group (GG) of index lesions and other tumour foci show that 10 patients had high-grade disease (defined as an index lesion with GG ≥ 3) and the remaining 9 patients had low-grade disease (defined as an index lesion with GG ≤ 2).

**Table 1:** Patient clinical, imaging and pathological details

Patient	PSA level (ng/mL)	Pathological T-stage	Prostate volume (cm^3^)	# Histology slices	GG (index lesion)	GG (other foci)	PIRADS v2 (index lesion)	SUVmax
11	6.8	T3b	36.89	4	3	3, 4	5	3.88
12	16	T3a	64.35	4	3	–	5	6.47
20	9	T3b	33.25	5	5	1, 2, 4	4	9.64
26	42	T3a	52.17	4	3	1	4	12.11
34	8.1	T3a	50.34	4	2	2	5	3.70
46	3.2	pT2	33.49	3	3	1, 2	4	3.08
51	18	pT2	54.08	6	2	1	5	5.04
53	10	pT3a	33.77	5	3	–	5	5.95
54	12	pT2	34.03	5	2	1	3	6.45
55	6.5	pT2	30.23	4	3	1, 2	5	14.78
56	6.6	pT2	41.07	5	2	2	5	2.43
58	5.6	pT3b	48.55	5	5	Mix of 1–5	5	15.76
59	10.7	pT3a	39.40	5	2	–	Indeterminate	10.91
60	6.7	pT3a	49.80	5	2	1	4	3.23
62	10.7	pT3a	31.81	4	5	1	5	46.34
64	9	pT3a	37.44	5	3	1, 2	5	59.40
65	7.3	pT2	35.69	4	2	1, 2	5	7.79
66	6.5	pT2	26.21	5	2	–	2	8.46
68	10.8	pT2	57.33	8	2	1, 2	5	5.75

Prostate volume is calculated using the ellipsoid formula from pathology measurements [11]

*GG* Grade group

### Multiparametric MRI

In vivo mpMRI was acquired using two 3 T scanners, the first five patients scanned with a Siemens MAGNETOM Trio (Siemens Healthcare GmbH, Erlangen, Germany) and all other patients scanned with a Siemens MAGNETOM Skyra. The imaging protocol followed guidelines from the European Society of Urogenital Radiology (ESUR) [12] and included T2-weighted (T2w), Diffusion-Weighted Image (DWI) and Dynamic Contrast-Enhanced (DCE) imaging. A surface body coil was used without an endorectal coil to reduce the chance of deformation of the prostate, and patients without contraindications were given Buscopan to reduce peristaltic motion. T2w imaging was obtained using a 2D turbo spin echo sequence using acquisition matrix = 320 × 320, FOV = 160 mm × 160 mm, slice thickness = 3 mm, TE = 89–96 ms, TR = 3500–4830 ms. DWI images were obtained using a 2D spin echo sequence with echo planar readout, with *b*-values = 50, 400, 800 and 1200 s/mm^2^, acquisition matrix = 250 × 250, FOV 250 mm × 250 mm, slice thickness = 4 mm. Apparent Diffusion Coefficient (ADC) maps were computed from DWI images using inline software.

Pre-contrast 3D T1-weighted images with variable flip angles (5°, 10°, 15°, 20°, 30°) were acquired. DCE-MRI was performed using a 3D spoiled gradient echo with a time-resolved view sharing sequence for high temporal resolution imaging (TWIST, Siemens Healthineers, Erlangen, Germany). Each patient received a 10-ml bolus injection of contrast agent Dotarem (gadoterate meglumine, Guerbet, USA), followed by a saline flush. Semi-quantitative parameters and pharmacokinetic parameters were computed using Dynamika software (Image Analysis Group, London, UK) [13]. Semi-quantitative parameters included the initial rate of enhancement (IRE), the time to peak enhancement (TTP), the maximum enhancement (ME), the time of contrast agent onset (Tonset), the time of contrast agent washout (Twashout) and the initial rate of washout (IRW). Pharmacokinetic parameters were computed using the Tofts model [14] including Ktrans (the volume transfer constant between blood plasma and extra-vascular extra-cellular space) and Ve (volume of extra-vascular extra-cellular space). The initial area under the gadolinium contrast agent concentration curve for the first 60 s post-injection (iAUGC60) was also computed.

### PET/CT imaging

All patients had PET/CT imaging after injection with a 68 Ga PSMA-HBED-CC (PSMA-11) tracer. Five different PET/CT scanners were used, with scanning from the base of the skull or the vertex to the upper thighs. The PET scanning bed steps were acquired starting at the upper thighs to minimise the chance of spatial shifts between PET and CT at the level of the prostate, which can be caused by patient movement, bladder filling or intestinal movements. Further acquisition details are given in Additional file 1: Table S1, including the PET image reconstruction method, Gaussian filter kernel size, the time of bed positions, tracer uptake time and PET and CT image resolution information. PET images were corrected for attenuation using the contemporaneous low-dose non-contrast CT scan and normalised by body weight to obtain PET Standardised Uptake Values (SUV) images for each patient.

### Ex vivo MRI and histology data

After prostatectomy, the prostate specimens were embedded in agarose gel in a custom-made sectioning box for ex vivo MRI scanning, after which the specimen was cut into 5-mm sections and then microtomed at 3 µm to obtain whole-mount haematoxylin and eosin (H&E) stained sections. The most apical and basal histology sections obtained were not included in this study, as standard pathology processing required them to be cut in a parasagittal orientation which could not be easily co-registered with ex vivo MRI. Table 1 details the number of axial histology sections obtained for each patient, and the estimated prostate volume calculated using the ellipsoid method and prostate specimen measurements [11]. Each axial H&E-stained section was annotated for tumour and assigned a GG by an experienced uro-pathologist (CM) and digitised with an Epson Perfection V700 scanner to give images approximately 0.01 mm resolution. Further details of this are given in Reynolds et al. [15].

### Co-registration

**Fig. 1:**
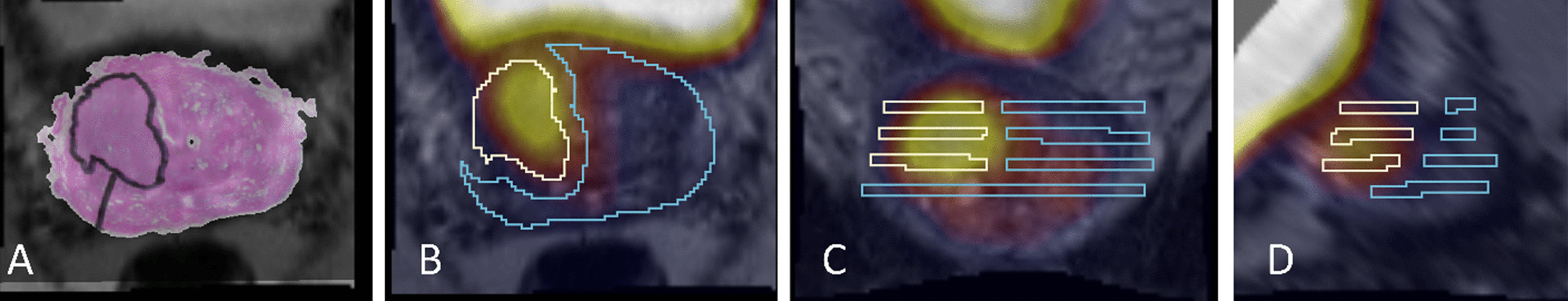
Co-registered imaging data for patient 26 showing **a** histology and T2w MRI with tumour annotated by a pathologist, and **b**–**d** axial, coronal and sagittal (respectively) T2w MRI aligned with PET showing tumour voxels defined from histology outlined in white and benign voxels defined as being 3.3 mm beyond the tumour voxel boundary outlined in blue

Co-registration of PET/CT with mpMRI and ground truth histology was carried out using our established framework [10, 15], which utilised ex vivo MRI to account for tissue deformation and shrinkage after prostatectomy. In brief, the PET and CT images were first qualitatively inspected to determine whether there were any spatial shifts between them due to the different timing between the scans; however, none of the PET or CT images required manual correction. Then, PET images were rigidly registered with in vivo 3D T2w MRI in 3D Slicer software [16] by using the contemporaneous CT, which provided anatomical information and higher resolution than the PET image. The computed transformation between CT and in vivo 3D T2w MRI was then applied to co-register PET with in vivo MRI. The 2D T2w, ADC maps from DWI and DCE MRI parameter maps were rigidly registered with the reference 3D T2w MRI in 3D Slicer. All co-registered in vivo MRI and PET/CT data were re-sampled to isotropic 0.8 mm voxels to match the 3D T2w MRI resolution, and deformable image registration was applied to co-register with ex vivo MRI and ground truth histology data. Figure 1 shows an example co-registered dataset with PET, in vivo T2w MRI and histology.

### Correlation analysis

Correlation analysis was carried out at a voxel-wise level using the co-registered images to investigate the relationship between signals on mpMRI and PET SUV values. For this and subsequent analyses, DCE MRI parameters Tonset, Twashout and IRW were excluded as the Tonset parameter was inconsistent across the dataset and challenging to reproduce, while Twashout and IRW contained many zero value pixels as the contrast agent had not washed out from the entire prostate during the imaging timeframe. To account for an estimated 3.3 mm average registration uncertainty between histology and in vivo mpMRI and PET/CT computed in our prior study [15], benign voxels were defined as all voxels within the prostate contour which were at least 3.3 mm away from the tumour boundary (see Fig. 1). Additionally, small tumour foci with an area below that for a tumour with an average 5 mm diameter were excluded, corresponding to the upper bounds of the registration uncertainty as well as studies indicating the minimum tumour size that can be identified on mpMRI [17, 18].

Kolmogorov–Smirnov tests were performed to determine whether PET SUV and mpMRI voxel values for tumour and benign tissue were normally distributed, and whether the benign and tumour voxel values for each imaging parameter exhibited the same distribution. Spearman correlation coefficients were computed to assess the degree of correlation between the PET SUV and the mpMRI parameter tumour voxel values. Bonferroni correction was applied to the correlation cut-off p-values to assess its significance.

### Feature extraction and selection

Voxel-wise radiomic features were extracted from co-registered mpMRI and PET imaging data using the PyRadiomics Library (v.3.0.1) [19] in the Python programming environment (v3.8). Features were extracted from the PET, T2w MRI, ADC maps from DWI and DCE MRI parameters TTP, Ktrans and iAUGC60. These DCE MRI parameters were chosen based on prior studies which indicated they were the most predictive for tumour [20–22]. Radiomic features included first-order statistics, shape-based and texture features based on Grey Level Co-occurrence Matrix (GLCM) (number of features, *n* = 24), Grey Level Run Length Matrix (GLRLM) (*n* = 16), Grey Level Size Zone Matrix (GLSZM) (*n* = 16), Neighbouring Grey Tone Difference Matrix (NGTDM) (*n* = 5), Grey Level Dependence Matrix (GLDM) (*n* = 14). Radiomic features were extracted from the original images and after applying filters including wavelet transform, gradient magnitude, Laplacian of Gaussian, 2D local binary pattern (LBP) and 3D LBP. A kernel size of 9 voxels in each direction (7.2 mm in each direction) was chosen, as it was closest to the average tumour radius in the dataset when assumed to be spherical.

Patient-based clinical features including age and PSA level, and PET-specific features were added to the model, including SUVmax, SUVmean, radioactive tracer uptake time and tracer radioactivity. Feature reduction was performed to prevent overfitting and reduce model training cost by applying the following strategies: (i) reject all highly correlated features, (ii) retain the top 10% of the features based on the ANOVA test and (iii) retain the top 50 features based on the mean decrease in random forest Gini impurity.

### Tumour detection and grading

Machine learning classifiers were trained to predict tumour location using a radial basis function kernel with the Python scikit-learn library (v0.24.2) [23]. Two classifiers were used to compare performance, a Random Forest Classifier (RFC) and a Support Vector Classifier (SVC). A fivefold cross-validation scheme was used to optimise the parameters of each classifier via a halving successive grid search procedure. Data imbalance, caused by the number of benign voxels vastly surpassing the number of tumour voxels, was addressed through data augmentation by flipping images 180 degrees about the axial, sagittal and coronal axes. This increased the number of tumour voxels fourfold.

Classifiers were trained to predict tumour location using (1) PET images alone, (2) mpMRI data alone (T2w, ADC, TTP, Ktrans and iAUGC60) and (3) mpMRI and PET images combined. For the best performing tumour detection model, a second model was trained to predict the grade of each predicted tumour voxel, by classifying it as either high grade (defined as GG ≥ 3) or low grade (defined as GG ≤ 2). The performance of the tumour grade prediction model was assessed using balanced accuracy and weighted F1 score. Balanced accuracy was computed via the arithmetic mean of the sensitivity and specificity, measuring the combined performance of the tumour detection and tumour grading models. The weighted F1 score was calculated by taking the average F1 score from the high-grade and low-grade grading performance, which were each weighted by the proportion of voxels with the corresponding grade in the patient.

## Results

### Correlation analysis

**Fig. 2:**
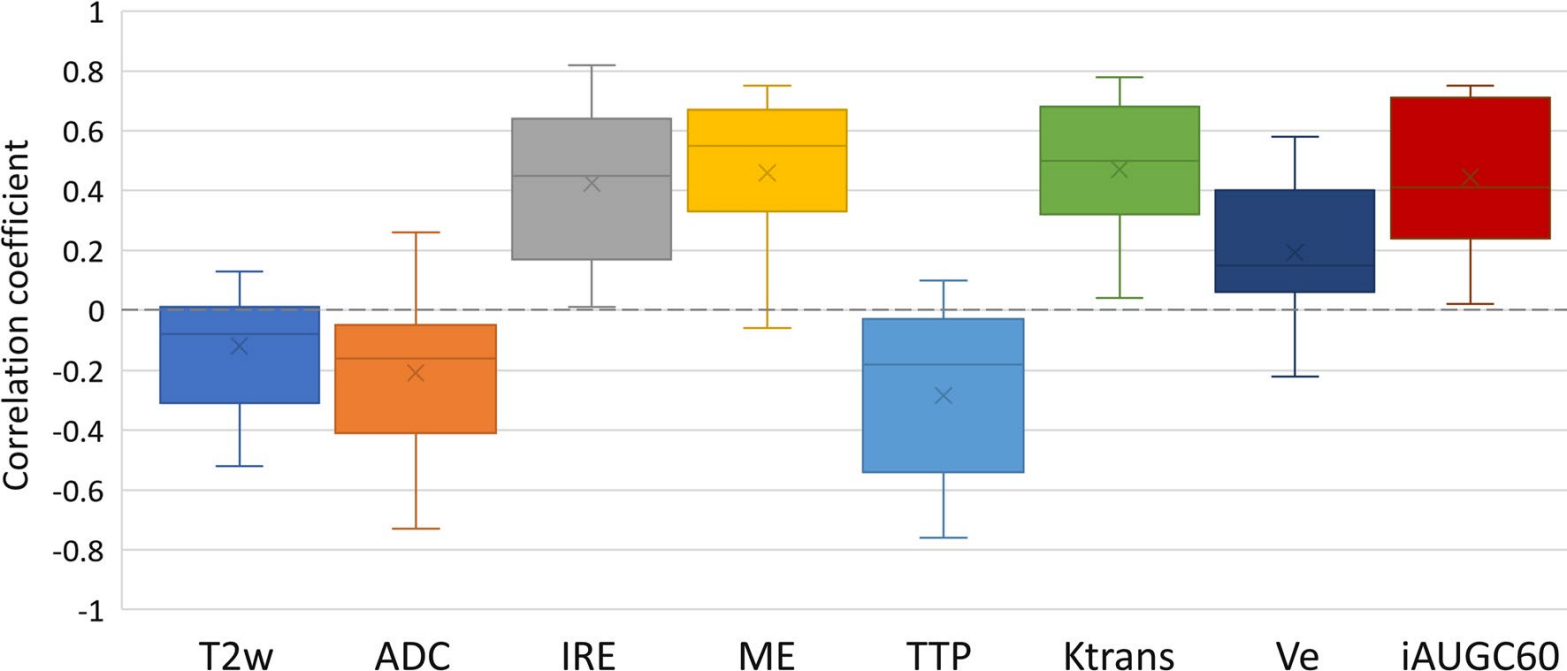
Box and whisker plot showing the range of Spearman correlation coefficient values computed between PET SUV and MRI parameter voxel values. Outlier values were excluded, and mean data values are shown with an ‘*x*’

Kolmogorov–Smirnov tests showed the distribution of voxel values for each imaging parameter did not follow a normal distribution for either tumour or benign tissue, and the distribution of all parameter values was significantly different between tumour and benign voxels. Figure 2 shows a box and whisker plot with the range of Spearman correlation coefficient values across all patients for each mpMRI parameter compared with PET SUV. Additional file 1: Table S2 gives more detailed Spearman correlation coefficient values (mean, standard deviation, median, minimum and maximum) with further separation into low-grade and high-grade tumours.

Overall, the parameter which was the most highly correlated with PET SUV was Ktrans (mean 0.470, range 0.040–0.780), followed by ME (mean 0.459, range -0.060 to 0.750) and then iAUGC60 (mean 0.445, range 0.020 to 0.750). For high-grade tumours, Ktrans remained the most highly correlated parameter (mean 0.546), followed by iAUGC60 (mean 0.552) and then ME (mean 0.504), whereas for low-grade tumours, ME was the most highly correlated parameter with PET SUV (mean 0.410), followed by Ktrans (mean 0.386) and then iAUGC60 (mean 0.326). The DCE MRI parameter IRE was positively correlated with PET SUV for all patients, and Ve was positively correlated for most voxels. The DCE MRI parameter TTP mainly showed negative correlations with PET SUV tumour voxel values (mean -0.284, range -0.760 to 0.100), whereas T2w and ADC did not show a high degree of correlation with PET SUV.

### Tumour detection

Table 2 details the cross-validation score (*F*_1_) for each tumour detection model: PET alone, mpMRI alone, and PET with mpMRI combined. The RFC performed better than the SVC for all three models, where the best performing model overall was shown by the combined PET and mpMRI RFC, which gave a cross-validation score of 0.757. Each RFC model was then re-trained using a patient-wise leave-one-out scheme to generate tumour location predictions for each patient.

**Table 2:** Cross-validation performance of the tumour detection models

Model	Cross-validation score (*F*_1_)	
	RFC	SVC
PET	0.652	0.534
mpMRI	0.716	0.543
PET + mpMRI	0.757	0.565

**Fig. 3:**
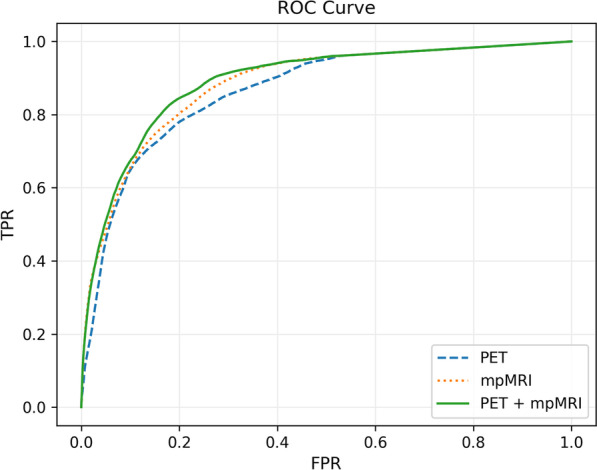
ROC curves for each of the tumour detection models. *TPR* True positive rate, *FPR* False positive rate

Table 3 details the individual patient sensitivity, specificity and Area Under the Receiver Operating Characteristic Curve (ROC-AUC) values for the three RFC tumour prediction models and the overall model performance. Patient 46 has been excluded from these results, as the patient’s tumour foci were smaller than the predetermined threshold. Figure 3 shows the plotted ROC-AUC curves for the overall model performance, which indicates the PET and mpMRI combined model gives a higher overall performance (sensitivity 0.842, specificity 0.804, ROC-AUC 0.890) than PET alone (sensitivity 0.781, specificity 0.799, ROC-AUC 0.865) or mpMRI alone (sensitivity 0.802, specificity 0.801, ROC-AUC 0.882). However, Table 3 also shows that the PET alone model or the MRI alone model can outperform the combined model for individual patients.

**Table 3:** Patient-wise leave-one-out performance of each RFC detection model and the overall performance

Patient	PET			mpMRI			PET + mpMRI		
	Sensitivity	Specificity	ROC-AUC	Sensitivity	Specificity	ROC-AUC	Sensitivity	Specificity	ROC-AUC
11	0.937	0.531	0.681	0.937	0.531	0.885	0.937	0.533	0.878
12	0.662	0.968	0.897	0.877	0.828	0.878	0.663	0.944	0.888
20	0.899	0.555	0.882	0.316	0.823	0.719	0.846	0.745	0.861
26	0.839	0.921	0.938	0.883	0.848	0.935	0.881	0.912	0.946
34	0.865	0.629	0.809	0.150	0.873	0.755	0.798	0.690	0.801
51	0.000	1.000	0.781	0.898	0.843	0.933	0.770	0.898	0.922
53	0.975	0.614	0.892	0.970	0.732	0.931	0.829	0.795	0.899
54	0.936	0.468	0.803	0.827	0.527	0.685	0.888	0.550	0.732
55	0.735	0.666	0.808	0.784	0.818	0.876	0.880	0.726	0.869
56	0.000	1.000	0.674	0.391	0.930	0.771	0.330	0.967	0.780
58	0.849	0.836	0.902	0.730	0.930	0.908	0.833	0.876	0.928
59	0.843	0.750	0.853	0.941	0.703	0.876	0.908	0.709	0.863
60	0.032	0.922	0.636	0.000	0.968	0.806	0.006	0.933	0.607
62	0.915	0.863	0.944	0.754	0.951	0.935	0.891	0.910	0.942
64	0.638	0.817	0.832	0.890	0.739	0.878	0.863	0.768	0.872
65	0.991	0.543	0.864	0.909	0.731	0.919	0.948	0.633	0.911
66	0.623	0.957	0.949	0.515	0.899	0.869	0.614	0.940	0.919
68	0.415	0.880	0.789	0.613	0.830	0.833	0.688	0.838	0.851
Overall	0.781	0.799	0.865	0.802	0.801	0.882	0.842	0.804	0.890

**Fig. 4:**
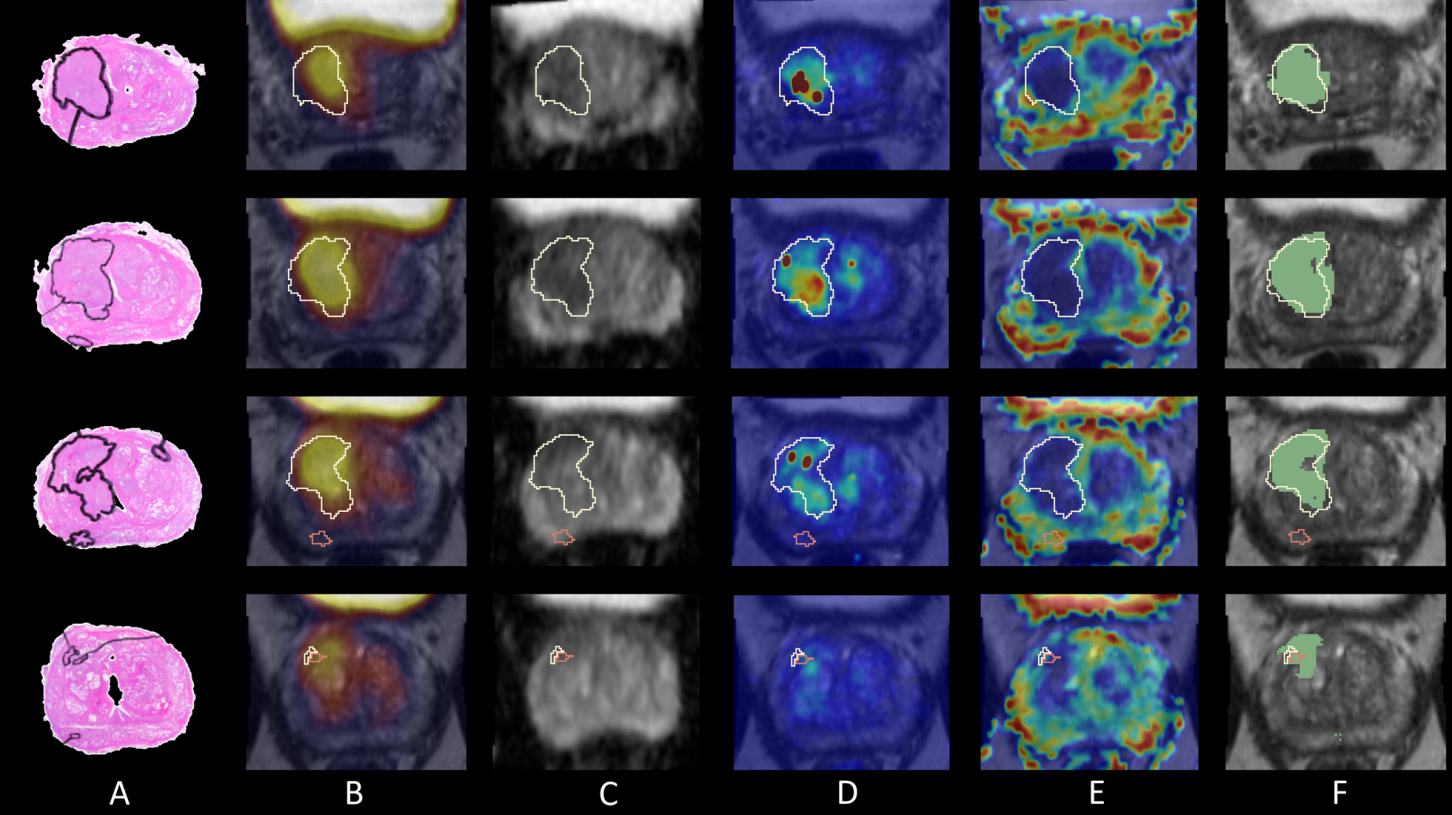
Results from the best performing patient 26 showing four image slices from top to bottom (base to apex) with: **a** tumour annotations on histology, **b** PET and T2w MRI, **c** the ADC map, **d** Ktrans map, **e** TTP map and **f** the predicted tumour voxels in green overlaid on the T2w MRI, each visualised with the tumour location outline from histology

Figure 4 shows co-registered images and the predicted tumour location (Fig. 4f) from the best performing patient 26, where the sensitivity was 0.881, specificity 0.912 and ROC-AUC 0.946. This patient had a high-grade GG3 (PIRADS 4) index lesion and a high PSA of 42 ng/mL. The tumour annotations from co-registered histology correspond well with the focal uptake shown on PSMA PET, a region of decreased ADC values, increased Ktrans values and decreased TTP values which are all typical within tumours. The predicted tumour location (Fig. 4f) was consistent with the tumour annotations from histology across all four slides, but missed a small tumour foci on the third slide which had low-grade GG1.

**Fig. 5:**
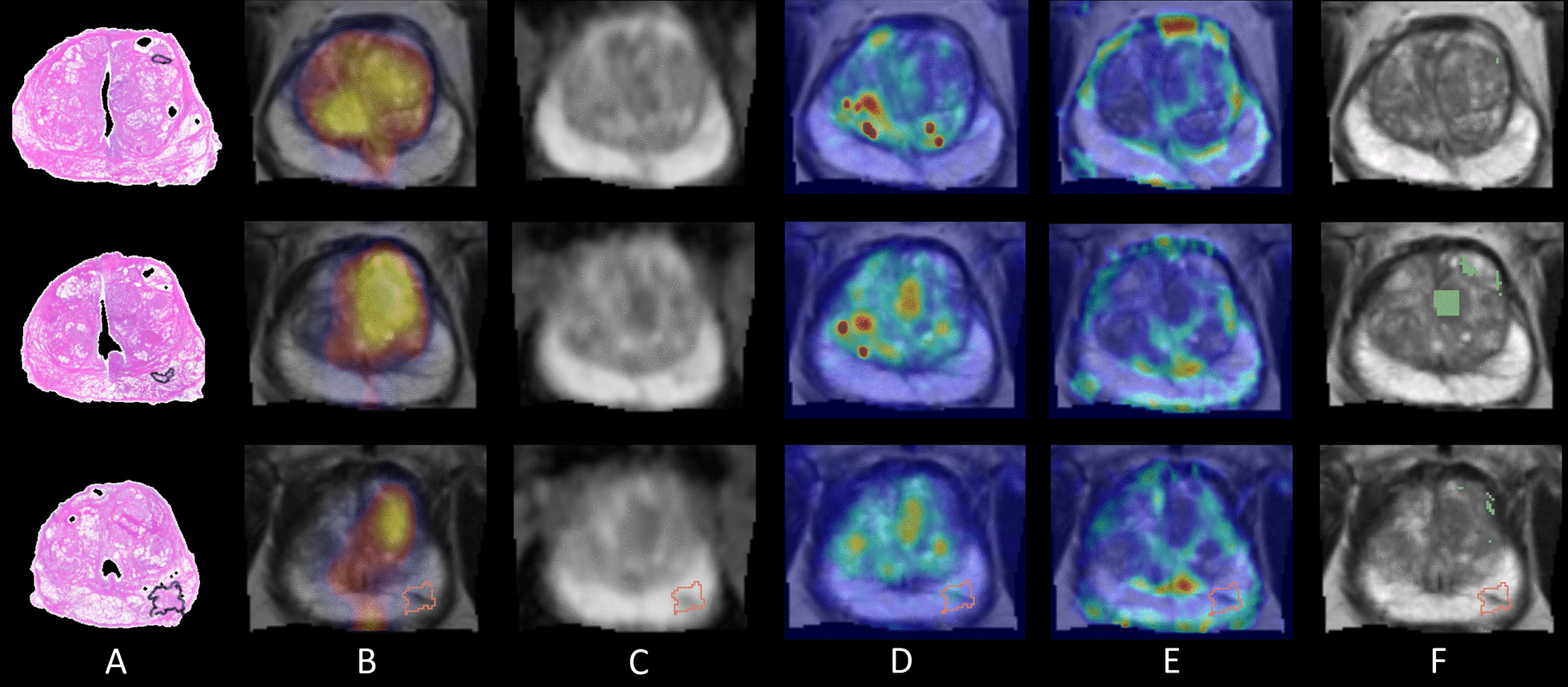
Results from the worst performing patient 60 showing three histology slices out of six, which had tumour visible, from top to bottom (mid-gland to apex) with: **a** tumour annotations on histology, **b** PET and T2w MRI, **c** the ADC map, **d** Ktrans map, **e** TTP map and **f** the predicted tumour voxels in green overlaid on the T2w MRI, each visualised with the tumour location outline from histology

Figure 5 shows co-registered images and the predicted tumour location from the worst performing patient 60, using the combined PET and mpMRI model, which gave sensitivity 0.006, specificity 0.933 and ROC-AUC 0.607. This patient had a PSA of 6.7 ng/mL and a low-grade index lesion of GG2 (PIRADS 4) on the left posterior apex along with other tumour foci with GG1 which were excluded from the analysis for being too small. Histopathology showed this patient had high-grade prostatic intraepithelial neoplasia (HG-PIN) within the prostate which appears to show as a region of decreased diffusion on ADC and corresponds to a region of high uptake on PET images. As a result, the tumour location is incorrectly predicted to be within this region of decreased ADC and increased PET signal while entirely missing the small index lesion.

**Fig. 6:**
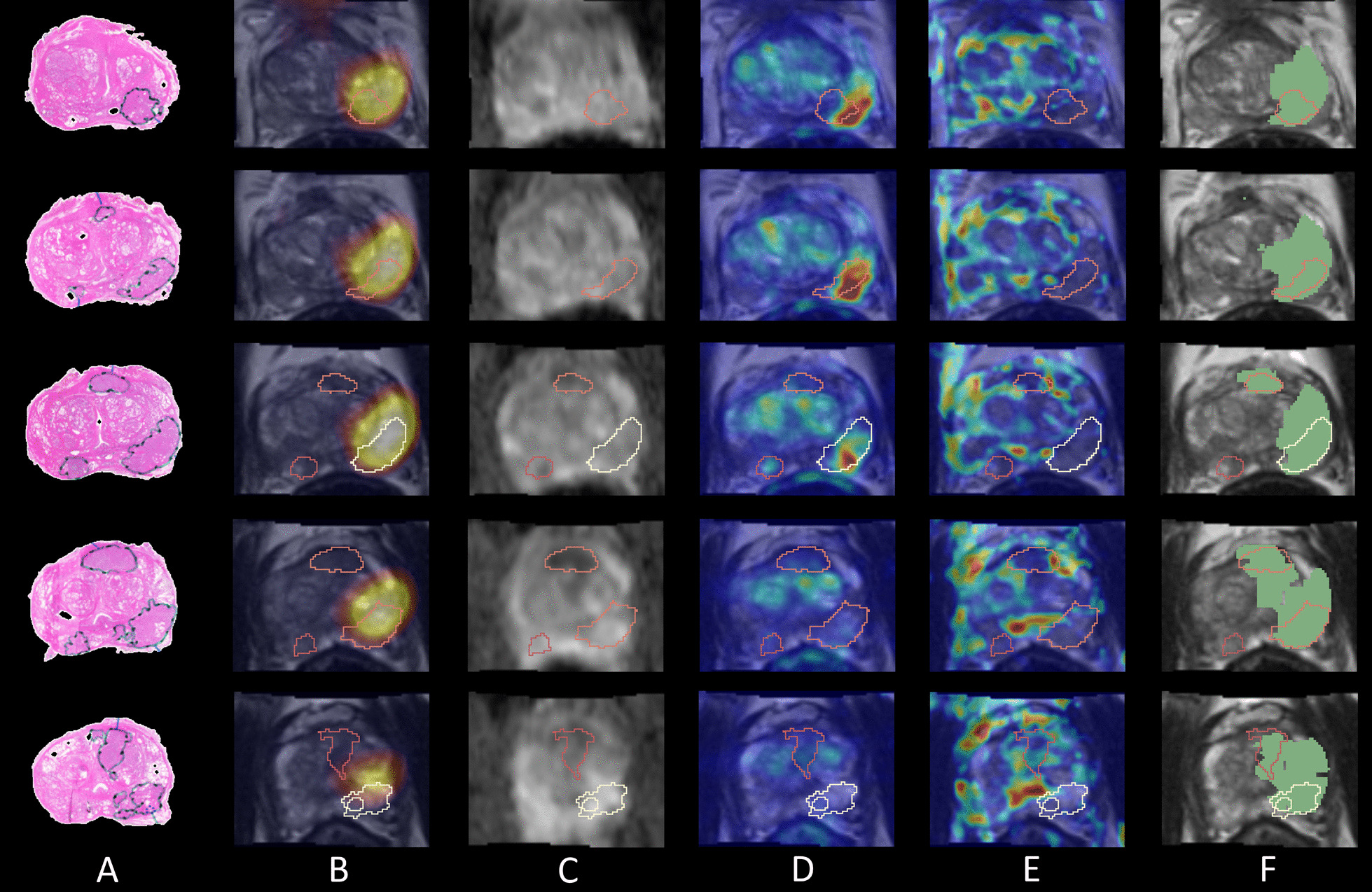
Results from patient 64 showing image slices top to bottom (base to apex) with **a** tumour annotations on histology, **b** PET and T2w MRI, **c** the ADC map, **d** Ktrans map, **e** TTP map and **f** the predicted tumour voxels in green overlaid on the T2w MRI, each visualised with the tumour location outline from histology

Results from an average performing patient (number 64) are shown in Fig. 6, where there were multiple tumour foci within the prostate. This patient had a high-grade index lesion of GG3 (PIRADS 5) in the left lobe posterolaterally, while other tumour foci were low-grade GG1 and GG2 lesions. The combined PET and mpMRI model was able to identify the index lesion which corresponded well to an area of high uptake on PET, increased Ktrans and decreased TTP. The model was also able to predict the low-grade GG2 tumour foci in the anterior mid zone (shown in the last three slices in Fig. 6f) but was unable to identify the lowest grade GG1 lesion in slices three and four. Overall, the tumour location is over predicted, which is reflected in the model sensitivity, specificity and ROC-AUC values of 0.863, 0.768 and 0.872.

The top 10 performing features in each of the RFC tumour detection models are listed in Table 4. The top three features in the combined model were all radiomic features from PET, after which the next two important features were radiomic features from ADC. SUVmax was the sixth most important feature in this model. For the PET alone model, the same 3D LoG radiomic feature was the most important followed by a mixture of clinical and PET radiomics-based features. The mpMRI model showed that PSA was more important than any of the other radiomic features, followed by ADC radiomic features and various TTP radiomic features from DCE MRI.

**Table 4:** Top 10 features in each of the tumour detection models ranked from most to least important

Rank	PET model		mpMRI model		mpMRI + PET model	
	Image	Feature	Image	Feature	Image	Feature
1	PET	3D LoG *σ* = 3 mm minimum	–	PSA	PET	3D LoG *σ* = 3 mm minimum
2	–	Uptake time	ADC	NGTDM Coarseness	PET	Gradient Magnitude Energy
3	PET	SUVmax	ADC	10^th^ percentile	PET	LBP 3D m = 1 maximum
4	–	PSA	TTP	10^th^ percentile	ADC	NGTDM Coarseness
5	PET	LBP 3D m = 1 maximum	ADC	GLCM Correlation	ADC	10^th^ percentile
6	PET	SUV	ADC	Entropy	PET	SUVmax
7	PET	Gradient Magnitude Energy	–	Age	ADC	GLCM Correlation
8	–	Age	ADC	GLDM LDLGLE	ADC	Entropy
9	–	Injected activity	TTP	90^th^ percentile	–	PSA
10	–	Grade Group other	TTP	GLDM LDLGLE	PET	GLDM LDLGLE

*LoG* Laplacian of Gaussian, *LBP* Local binary pattern, *NGTDM* Neighbouring Grey Tone Difference Matrix, *GLDM* Grey Level Dependence Matrix, *LDLGLE* Large Dependence Low Grey Level Emphasis

### Tumour grading

Tumour classification was carried out for each patient, to classify whether a predicted tumour voxel was high grade or low grade, using output from the combined PET and mpMRI model. This tumour grading model was built using an RF classifier, and a patient-wise leave-one-out cross-validation scheme, to give the performance results as shown in Table 5. The overall accuracy of the tumour grading model ranged from 0.671 to 0.992, and low-grade balanced accuracy was from 0.496 to 0.863, while high-grade balanced accuracy was 0.500 to 0.891. The weighted F1 score had a relatively low range from 0.000 to 0.486 with the worst result for patient 60 who had no correct tumour predicted (see Fig. 5f), while the best result was for patient 26 whose tumour location prediction was the best from all patients (see Fig. 4f).

**Table 5:** Overall performance of tumour grading RFC model, with patients ordered according to whether they had a low-grade (LG) or high-grade (HG) index lesion

Patient	Grade	Low-grade balanced accuracy	High-grade balanced accuracy	Overall accuracy	Weighted *F*1 score
34	LG	0.767	–	0.841	0.143
51	LG	0.705	–	0.975	0.329
54	LG	0.760	–	0.802	0.201
56	LG	0.530	–	0.984	0.092
59	LG	0.844	–	0.827	0.274
60	LG	0.496	–	0.992	0.000
65	LG	0.863	0.500	0.906	0.222
66	LG	0.687	–	0.964	0.211
68	LG	0.723	–	0.892	0.143
11	HG	–	0.786	0.671	0.338
12	HG	–	0.636	0.969	0.100
20	HG	0.500	0.756	0.950	0.145
26	HG	0.495	0.858	0.977	0.486
53	HG	0.783	0.500	0.905	0.015
55	HG	0.758	0.504	0.905	0.114
58	HG	0.500	0.891	0.958	0.242
62	HG	0.554	0.849	0.927	0.282
64	HG	0.730	0.581	0.922	0.134

## Discussion

In this study we have analysed the voxel-wise relationship between PSMA PET SUV and mpMRI parameters and developed radiomics-based machine learning models to predict tumour location and grade. This builds upon our earlier proof-of-concept study [10] which had a smaller cohort of nine patients imaged with a non-uniform set of PET tracers with only five having the Ga68-PSMA-11 tracer. For this study, we combined imaging from these five patients with an additional 14 patients who all had PET imaging with the Ga68-PSMA-11 tracer, along with mpMRI according to ESUR guidelines [12], to ensure data consistency. Furthermore, the earlier study did not investigate the use of radiomics-based machine learning models to predict tumour location and grade which we have been able to develop here, for the future goal of implementing BiRT which requires a voxel-level dose distribution.

Strengths of this study include the use of a highly controlled dataset, the accurate co-registration of PET/CT and mpMRI with ground truth histology data using an established framework [15], and the inclusion of DCE MRI parameters when most other studies have incorporated only ADC and T2w imaging from mpMRI. Furthermore, the voxel-wise approach differs from most other studies which have used a region of interest (ROI)-based approach, and the step-wise development of two classifiers to predict tumour location and then tumour grade is unique, as all other studies, according to our knowledge, have focussed on only one of these classification tasks.

### Correlation analysis

Correlation analysis was conducted using Spearman rank correlation between imaging parameters rather than Pearson correlation, which had been used in our prior study [10]. The Spearman rank correlation method was considered more appropriate, as it did not assume the underlying data were normally distributed which was confirmed by the Kolmogorov–Smirnov test results. Despite the different correlation method, overall correlation trends validated our earlier findings [10], confirming that perfusion-related mpMRI parameters from DCE MRI are most strongly correlated with PSMA PET SUV, whereas ADC and T2w MRI are not strongly correlated. Quantitative parameters Ktrans and iAUGC60, the most common DCE MRI biomarker used in oncology trials [22], and semi-quantitative parameters ME and IRE each showed consistently strong positive correlations with PSMA PET SUV, while the semi-quantitative parameter TTP showed a negative correlation with PSMA PET SUV. These findings are consistent with a study by Zhao et al. [20] who compared PSMA PET with DCE MRI parameters in 39 patients and reported malignant lesions had significantly shorter TTP than benign lesions. Overall results indicate that tumour voxels with increased PSMA PET tracer uptake correspond with higher levels of tissue perfusion, a key characteristic of tumours.

### Tumour detection

Many artificial intelligence models that use radiomic features have been developed to predict tumour location from prostate mpMRI, with some gaining FDA and CE approval [24, 25]. In contrast, relatively few studies have predicted tumour location or tumour grade using radiomic features from PET and mpMRI in combination [26]. This is partially due to a lack of standardisation for PET imaging and for computing PET radiomic features, as well as limited datasets available with ground truth histopathology for model development and validation. The larger voxel sizes used in PET and inherently lower signal-to-noise ratio when compared with mpMRI also provide challenges [27]. Standard PET SUV parameters, including SUVmax, are often used in clinical practice however PET radiomic features offer significant potential for prostate cancer applications [27] which may outperform standard metrics, and studies using PET radiomics are on the rise [28–32].

The mpMRI and PET radiomics-based models developed in this study, contribute towards this growing research field. The tumour prediction and tumour grading models were developed as two separate classification tasks, with the predicted tumour voxels output by the tumour detection model being further classified into high-grade or low-grade voxels by the grading classifier. A single classifier approach would have resulted in a multi-class classification problem, with three classes: benign, high-grade tumour and low-grade tumour, which would have given a highly imbalanced class split in the dataset requiring a lot of data augmentation to reduce the imbalance and more feature inputs. Hence the two-classifier approach was preferred as it lowered the computational cost and simplified the performance assessment for each task.

Results showed that an RFC model for detecting prostate cancer using combined PET and mpMRI radiomic features performed better than RFC models developed using radiomic features from either modality alone. This was not unexpected, due to the aforementioned complementary nature of the imaging modalities as demonstrated in many non-radiomics studies and clinical trials [9, 33–35]. This result was also consistent with studies by Zamboglou et al. [36] and Spohn et al. [37] who did not utilise radiomics, but similarly validated imaging with ground truth pathology data and showed that PSMA PET is a valuable addition alongside mpMRI for defining the gross tumour volumes (GTV) for focal therapy applications, where mpMRI is more likely to underestimate tumour volume than PSMA PET [36, 37].

The top 10 features for each model in our study demonstrated the superior performance of PET radiomic features for tumour detection, compared to mpMRI radiomic features, and their better predictive performance than the commonly used metric SUVmax. When assessing the most predictive features from mpMRI, the ADC map ranked the highest in the combined and the mpMRI alone models, with the NGTDM coarseness texture feature being the top-ranking in both followed by the 10^th^ percentile ADC value. The only other mpMRI parameter with radiomic features in the top 10 list for the mpMRI alone model was the semi-quantitative DCE MRI parameter TTP, demonstrating the importance of perfusion imaging for detecting tumours especially when PSMA PET is unavailable.

### Tumour grading

The tumour grading model in our study showed promising results, with high overall accuracy values across patients. The performance of the grading model was limited by the tumour detection model however, because any voxel with undetected tumour would automatically be considered benign and the tumour grading model would not classify them into high grade or low grade at all. Therefore, further development of these models with larger datasets would be required to improve individual patient performance.

Several studies aiming to assess tumour grade using PET and mpMRI data can be compared with these findings. Domachevsky et al. [38] previously analysed data from 22 patients to characterise prostate cancer and cell density using Ga68-PSMA-PET/MRI data. While they did not extract radiomic features, they showed that PET SUVmax, ADCmin and ADCmean were distinct biomarkers for differentiating between tumours with Gleason Score ≥ 7 and benign tissue. In another study by Papp et al. [39], data from 52 patients were used to investigate the diagnostic performance of RFC classifiers with radiomic features from PSMA PET, ADC and T2w MRI to predict low-risk versus high-risk lesions. Their radiomics-based RFC model was better for predicting lesion risk than SUVmax (AUC was 0.86 versus 0.80). Their feature ranking analysis similarly showed that PSMA radiomic features were the most important, compared to ADC and T2w MRI features. A study by Solari et al. [29] reported the complementary value of PSMA-PET and ADC radiomics. With a retrospective cohort of 101 patients, they extracted radiomic features from the entire prostate gland and developed a series of SVM models using single modality and combined modalities to predict Gleason Score. Models which combined PET and ADC radiomic features outperformed single modality radiomics-based models and other combined modality radiomic-based models (PET + T1w and PET + T2w) to give balanced accuracy 82% ± 5%. In a recent study by Feliciani et al. [31], preliminary results were shown using radiomic features from PSMA PET and ADC maps to predict ISUP grade obtained from ground truth histology, which showed the complementary nature of PET and ADC radiomic features. In contrast to our study, none of these studies incorporated perfusion parameters from DCE MRI and they all had the tumour location delineated manually prior to development of their tumour grade model.

There are limitations to our study, including the small dataset of 19 patients, with imaging performed on two different MRI scanners and five different PET/CT scanners (Additional file 1: Table S1). Fourteen of 19 PET scans in the dataset were from two GE scanner types, which used the same VPFXS reconstruction method and PET voxel size of 2.86 mm × 2.86 mm × 3.27 mm; however, the time of bed positions varied between patients from 2 to 4 min. The remaining five patients in the dataset were scanned with three different Siemens scanner types and used either a point spread function (PSF) or and ordered-subset expectation maximisation (OSEM) reconstruction method with varying sized Gaussian filter kernels, differing bed position times ranging from 2 to 3.5 min and all utilised a larger in-plane resolution than GE scanner PET images. Each of these acquisition parameters can impact the partial volume effects in the PET images; however, in this study, it was not possible to account for all these variations and we assumed the images and SUV values could be directly compared. All PSMA PET and mpMRI parameter maps were resampled into 0.8-mm isotropic voxels to enable accurate co-registration with histology, which inherently assumed that resampling did not result in information loss or negatively impact radiomic feature extraction. Further studies would be required to determine how best to account for the partial volume effects caused by differing PET acquisition parameters for optimal utilisation in radiomic-based machine learning models.

Additional limitations include that histology data were not obtained from prostate tissue at the apex or the base, due to standard histology processing requiring these sections to be cut in a parasagittal manner which meant they could not be co-registered with imaging data. This means the spatial coverage of the histology used for ground truth validation varied between patients, with a median 5 histology slices covering 2 cm of tissue and ranging from a minimum 3 slices (covering 1 cm of tissue) and maximum 8 slices (covering 3.5 cm). Hence the predictive models may not be as accurate at the apex and base, as they are at the mid-gland. Only a selection of perfusion parameters from DCE MRI was used to develop the classification models; however, incorporating other parameters, such as Ve, may have improved accuracy. In addition, the prostate was not separated into peripheral and transitional zones, which would have allowed the development of zone-specific models as the tissue differs between zones.

There are no standardised rules for choosing kernel size, so a large kernel of 9 voxels in each direction was chosen to match the average tumour size on PET imaging; however, a different kernel size may have given better results and improved the detection of small tumours. Studies by Yi et al. [40] and Zamboglou et al. [32] may be valuable to consider here, as they have both recently demonstrated that PSMA PET radiomic features can detect invisible tumour lesions with high accuracy. Both researchers extracted radiomic features within the whole or half gland, but could not indicate where these invisible lesions were located, an important requirement for BiRT treatment planning.

## Conclusions

Machine learning models which utilise radiomic features from both PSMA PET and mpMRI in combination are better for predicting tumour location than using radiomic features from either modality alone. Model predictions could be used to inform voxel-wise dose distributions for biologically targeted radiation therapy treatment planning. Further work is warranted to externally validate such models and to improve their accuracy for classifying predicted tumour voxels into low-grade or high-grade disease to inform appropriate treatment strategies.

## Supplementary Information

**Additional file 1. Table S1**. PET/CT acquisition details. TOF Time of flight, PSF Point spread function, OSEM Ordered-subset expectation maximization. Note the Gaussian filter kernel size was not availablein the DICOM header for GE scanners. **Table S2**. Spearman correlation coefficients between PET SUV and MRI parameters.

## Data Availability

The dataset and code used during the current study are available from the corresponding author on reasonable request.

## Abbreviations

ADC: Apparent Diffusion Coefficient
BiRT: Biologically targeted Radiation Therapy
DCE: Dynamic Contrast-Enhanced
DWI: Diffusion Weighted Imaging
ESUR: European Society of Urogenital Radiology
GG: Gleason Grade
GLCM: Grey Level Co-occurrence Matrix
GLDM: Grey Level Dependence Matrix
GLRLM: Grey Level Run Length Matrix
GLSZM: Grey Level Size Zone Matrix
H&E: Haematoxylin and Eosin
HG: High Grade
iAUGC60: Initial Area Under the Gadolinium contrast agent concentration Curve for the first 60 s after injection
IPL: Intra-Prostatic Lesion
IRE: Initial Rate of Enhancement
IRW: Initial Rate of Washout
Ktrans: Volume transfer constant
LBP: Local Binary Pattern
LoG: Laplacian of Gaussian
LG: Low Grade
ME: Maximum Enhancement
mpMRI: Multiparametric MRI
NGTDM: Neighbouring Grey Tone Difference Matrix
PET/CT: Positron Emission Tomography/Computed Tomography
PI-RADS: Prostate Imaging-Reporting and Data System
PSA: Prostate-Specific Antigen
PSMA: Prostate-Specific Membrane Antigen
RFC: Random Forest Classifier
ROC-AUC: Area Under the Receiver Operating Characteristic Curve
ROI: Region Of Interest
SVC: Support Vector Classifier
T2w: T2-weighted
Tonset: Time of contrast agent onset
TTP: Time To Peak
Twashout: Time of contrast agent washout
SUV(max)(mean): Standardised Uptake Values (maximum) (mean)
Ve: Volume of extra-vascular extra-cellular space
