# Supplementary material for: Detecting localised prostate cancer using radiomic features in PSMA PET and multiparametric MRI for biologically targeted radiation therapy
Source: EJNMMI Res. 2023 Apr 26;13:34. doi: 10.1186/s13550-023-00984-5 (PMC10133419; doi:10.1186/s13550-023-00984-5)
Supplement: Supplementary file 1 — Additional file 1. Table S1. PET/CT acquisition details. TOF Time of flight, PSF Point spread function, OSEM Ordered-subset expectation maximization. Note the Gaussian filter kernel size was not availablein the DICOM header for GE scanners. Table S2. Spearman correlation coefficients between PET SUV and MRI parameters. [file 13550_2023_984_MOESM1_ESM.pdf]

Supplementary Tables

Table S1: PET/CT acquisition details. TOF = time of flight, PSF = point spread function, OSEM = ordered-subset expectation maximization. Note the Gaussian filter kernel size was not available (n/a) in the DICOM header for GE scanners.

Patient	PET/CT scanner	Reconstruction method	Gaussian filter kernel size (mm)	Time of bed positions (min)	PET voxel size (mm^3)	CT voxel size (mm^3)	Injected Activity (Mbq)	PET uptake time (mins)
11	GE 690 with TOF	VPFXS	n/a	3	2.86 x 2.86 x 3.27	1.07 x 1.07 x 3.27	118	32
12	GE 690 with TOF	VPFXS	n/a	3	2.86 x 2.86 x 3.27	1.07 x 1.07 x 3.27	98	60
20	Siemens Biograph 64	PSF 3i21s	5	3.5	3.93 x 3.93 x 3.00	1.07 x 1.07 x 3.00	105	54
26	Siemens Biograph 64	PSF 3i21s	5	3	3.93 x 3.93 x 3.00	1.07 x 1.07 x 3.00	128	50
34	Siemens Biograph 16	PSF 3i14s	8	3	4.07 x 4.07 x 3.00	0.98 x 0.98 x 2.00	146	72
46	GE 710 with TOF	VPFXS	n/a	2	2.86 x 2.86 x 3.27	1.07 x 1.07 x 3.27	196	59
51	GE 710 with TOF	VPFXS	n/a	2	2.86 x 2.86 x 3.27	1.07 x 1.07 x 3.27	219	54
53	Siemens Biograph 20 mCT	OSEM 3D+TOF 2i21s	7	3.5	4.07 x 4.07 x 3.00	0.98 x 0.98 x 2.00	181	57
54	GE 710 with TOF	VPFXS	n/a	2	2.86 x 2.86 x 3.27	1.07 x 1.07 x 3.27	240	49
55	GE 710 with TOF	VPFXS	n/a	2.5	2.86 x 2.86 x 3.27	1.07 x 1.07 x 3.27	194	48
56	GE 710 with TOF	VPFXS	n/a	2.5	2.86 x 2.86 x 3.27	1.07 x 1.07 x 3.27	153	58
58	GE 710 with TOF	VPFXS	n/a	3.5	2.86 x 2.86 x 3.27	1.07 x 1.07 x 3.27	105	72
59	GE 690 with TOF	VPFXS	n/a	3.5	2.86 x 2.86 x 3.27	1.07 x 1.07 x 3.27	139	74
60	GE 690 with TOF	VPFXS	n/a	2.5	2.86 x 2.86 x 3.27	1.07 x 1.07 x 3.27	157	46
62	GE 710 with TOF	VPFXS	n/a	4	2.86 x 2.86 x 3.27	1.07 x 1.07 x 3.27	109	60
64	GE 690 with TOF	VPFXS	n/a	2	2.86 x 2.86 x 3.27	1.07 x 1.07 x 3.27	197	48
65	Siemens Biograph 16	PSF 3i14s	8	2	4.07 x 4.07 x 3.00	0.98 x 0.98 x 2.00	95	71
66	GE 710 with TOF	VPFXS	n/a	3.5	2.86 x 2.86 x 3.27	1.07 x 1.07 x 3.27	104	48
68	GE 690 with TOF	VPFXS	n/a	4	2.86 x 2.86 x 3.27	1.07 x 1.07 x 3.27	79	53

Table S2: Spearman correlation coefficients between PET SUV and MRI parameters.

	LG					HG					Overall				
	**Mean**	**SD**	**Median**	**Min**	**Max**	**Mean**	**SD**	**Median**	**Min**	**Max**	**Mean**	**SD**	**Median**	**Min**	**Max**
**T2w**	-0.182	0.187	-0.140	-0.520	0.110	-0.063	0.167	-0.045	-0.340	0.130	-0.119	0.182	-0.080	-0.520	0.130
**ADC**	-0.236	0.163	-0.160	-0.540	-0.050	-0.184	0.341	-0.155	-0.730	0.260	-0.208	0.266	-0.160	-0.730	0.260
**IRE**	0.340	0.214	0.370	0.010	0.620	0.501	0.277	0.615	0.090	0.820	0.425	0.256	0.450	0.010	0.820
**ME**	0.410	0.207	0.380	0.030	0.690	0.504	0.264	0.605	-0.060	0.750	0.459	0.237	0.550	-0.060	0.750
**TTP**	-0.202	0.231	-0.180	-0.540	0.100	-0.358	0.310	-0.360	-0.760	0.000	-0.284	0.280	-0.180	-0.760	0.100
**Ktrans**	0.386	0.225	0.430	0.040	0.650	0.546	0.233	0.660	0.180	0.780	0.470	0.237	0.500	0.040	0.780
**Ve**	0.239	0.222	0.150	-0.130	0.540	0.151	0.248	0.145	-0.220	0.580	0.193	0.234	0.150	-0.220	0.580
**iAUGC60**	0.326	0.249	0.350	0.020	0.660	0.552	0.218	0.675	0.230	0.750	0.445	0.254	0.410	0.020	0.750
